# Influence of the Type of Topical Fluoride Delivery at Various Restoration Time Points on the Micro-Shear Bond Strength of a Resin-Based Composite on Bleached Tooth Enamel

**DOI:** 10.7759/cureus.25811

**Published:** 2022-06-10

**Authors:** Shreya Khanna, Aditi Sarin, Amit Kumar Mishra, Jaimesh Kumar Bhagat, Amrita Kumari, Hasmukh Gosai

**Affiliations:** 1 Department of Conservative Dentistry and Endodontics, Government Dental College, Jamanagar, IND; 2 Department of Conservative Dentistry and Endodontics, Dentcraft Dental Clinic, Noida, IND; 3 Department of Prosthodontics, Vananchal Dental College and Hospital, Garhwa, IND; 4 Department of Conservative Dentistry and Endodontics, Mithila Minority Dental College and Hospital, Darbhanga, IND; 5 Department of Conservative Dentistry and Endodontics, Vananchal Dental College and Hospital, Garhwa, IND; 6 Department of Conservative Dentistry and Endodontics, Siddhpur Dental College and Hospital, Gandhinagar, IND

**Keywords:** bond strength, fluoride, dental adhesives, mcinnes solution, tooth bleaching

## Abstract

Background

This study aimed to evaluate the micro-shear bond strength (mSBS) of an adhesive applied to bleached enamel to determine the effects of fluoride supply and restoration time on the mSBS.

Methodology

In this study, we bleached 130 samples of enamel and split them into the following three groups of 40 each: group MI: McInnes bleaching solution; group MIF: McInnes bleaching solution + topical acidulated phosphate fluoride gel; group FMI: 2% fluoridated McInnes bleaching solution. Non-bleaching or fluoridation was performed on a group of 10. Subgroups were created for each group (except for the control) to be restored for seven, 14, or 21 days. The mSBS test was performed on a universal testing machine after Tygon tubes were filled with composite resin and put on enamel surfaces. Tukey’s post-hoc test (p = 0.05) and two-way analysis of variance were employed to analyze the data.

Results

The mSBS values obtained for all groups immediately and after seven days were lower, while at 14 and 21 days were similar to the control group. According to the data, group FMI had greater mSBS levels than groups MI and MIF, both immediately and seven days later.

Conclusions

When in-office bleaching was employed, only the fluoride McInnes solution was successful in quickly correcting the adverse effects of low mSBS.

## Introduction

In this new era of aesthetic dentistry, tooth whitening is a well-known, effective, quick, and safe procedure for the treatment of darkened unesthetic teeth [1]. In-office bleaching is a fantastic option for people who are unable to use at-home bleaching or want to achieve satisfactory results immediately [2]. In-office therapy, on the other hand, has been shown to produce higher postoperative tooth sensitivity [3-5].

Bleaching causes changes in the surface morphology of enamel, including erosion, porosity, and mineral content loss [6,7]. Enamel demineralization can be the cause that occurs due to pH lower than 5.2-5.8 [8]. Because of the altered demineralization/remineralization balance brought on by an increase in enamel acid exposure during bleaching, less enamel surface hardness and toughness to fracture occur [9]. The binding strength values are similarly lowered when the restoration is performed immediately after bleaching. A seven-day treatment period is a sufficient time for the peroxide to completely leach from the enamel that has been exposed to a 35% hydrogen peroxide solution; however, studies have shown that the bond strength must be restored by waiting for at least 14 or 21 days [10-14]. Tooth shape and position can be improved using composite veneers, although this is a more time-consuming procedure for many individuals. Additionally, removing the surface layer of enamel [15], treating enamel with alcohol [16], using organic solvent-based adhesives [17], antioxidants and desensitizing agents, and fluoride are some of the other suggested techniques to lessen the lower tooth-resin bond strength. Fluoride, whether present in the bleaching formulation or not, functions as a remineralizing agent, allowing inorganic material lost during the bleaching process to be recovered [18]. The McInnes bleaching agent was chosen for this study because it is one of the most regularly utilized bleaching agents in clinical settings for treating dental fluorosis. It was recommended primarily because of its superficial aspect, ease of manipulation, and low cost [4].

Few studies have examined whether fluoride can affect bond strength after a dental substrate has been bleached and have reported mixed results [13]. An examination into the effects of fluoride administration on the micro-shear bond strength (mSBS) values of an adhesive placed on McInnes bleached enamel has not been done yet. This is one of the goals of the current study. An adhesive applied on in-office McInnes-bleached Msb enamel was also investigated in this study, as were the effects at different time intervals (immediately, seven days, 14 days, and 21 days). Adhesive applied on McInnes-bleached enamel at the dental clinic was also examined over time to see if it affected mSBS (immediately, seven days, 14 days, and 21 days). The following null hypotheses were tested: the restoration time point and type of fluoride delivery and whether it will influence the mSBS values of the adhesive applied on bleached enamel.

## Materials and methods

Specimen preparation

After the Institutional Review Board of MP Shah Government Medical College approved the study (64/02/2022), 130 maxillary and mandibular anterior teeth were chosen for the study based on the stipulated criteria. The criteria included permanent non-carious teeth that were not more than 30 days old and were advised for extraction due to orthodontic therapy or periodontal disease.

The samples required at least 30 days of storage in deionized water at 4°C and an autoclave at 121°C for at least five minutes of ultrasonic cleaning. A quick gem pod and a continuous water system removed the foundations of their cemento-veneer junction around 2 mm below the surface. It was cleaned using an adjustable cup and a slow-speed handpiece that used oil-free, non-fluoridated fine pumice and water before dying. The samples were arranged on a piece of two-sided tape, put on a glass slide, and then implanted into polyvinyl chloride (PVC) tubes using self-fix acrylic sap with their labial sides facing upward according to the manufacturer’s instructions.

Experimental design

A total of 121 specimens were divided into the following three groups (n = 40) based on the bleaching treatment: group Mcinnes (MI) (n = 40) was bleached with McInnes solution without sodium fluoride, group McInnes fluoride (MIF) (n = 40) was bleached with McInnes solution followed by separate topical fluoride application with acidulated phosphate fluoride (APF) gel, and group fluoridated McInnes (FMI) (n = 40) was bleached with fluoridated McInnes solution.

A fourth control group was added with a sample size of 10. These 10 specimens were used as a control group and were not bleached or fluoridated. Further, according to the restoration time points, each of the former three groups (n = 40) was further divided into the following four subgroups (n = 10): immediately after bleaching, seven days, 14 days, and 21 days after bleaching.

Bleaching and fluoridation procedure

One milliliter of 36% hydrochloric acid, 1 mL of 30% hydrogen peroxide, and 2 mL of anesthetic ether were mixed in a dip dish at a ratio of 5:5:1 before being injected into the skin.

Fluoridated McInnes bleaching solution was also freshly prepared before application by mixing 5 mL of 36% hydrochloric acid, 5 mL of 30% hydrogen peroxide, and 1 mL of anesthetic ether at the ratio of 5:5:1 in a dappen dish. Subsequently, 10 mL of this solution was pipetted in a clean glass beaker. Then, 0.2 g of sodium fluoride was weighed on a digital weighing scale and added to the premeasured 10 mL of freshly prepared McInnes solution.

Groups MI and MIF were bleached with McInnes, while group FMI was bleached with the fluoridated McInnes solution. It took five minutes of unidirectional application of the bleaching chemicals to the enamel surface, with the solution replenished every 30 seconds using a cotton applicator. Afterward, it was dipped in deionized water for 30 seconds and blotted with an absorbent paper to dry.

After drying, group MIF was treated with APF gel for five minutes, cleaned with an absorbent filter paper, washed under de-ionized water after 30 minutes, and damped dry with the absorbent paper again. The samples were then subjected to the restorative procedure according to the subgroup of the sample.

Restorative procedure

Subgroups were recovered at the same time as the control group samples and were maintained at 37°C in artificial saliva. The artificial saliva was prepared by adding calcium chloride (0.22 g), sodium phosphate (1.07 g), sodium bicarbonate (1.68 g), and sodium azide (2 g) (weighed on a digital weighing scale) to 1 L of distilled water.

For the restoration process, the specimens were etched for 30 seconds with 37% phosphoric acid gel, washed for 30 seconds with de-ionized water, and dried for the same length of time with oil-free air spray. It took 20 seconds for each application of the Single Bond 2, 3M ESPE adhesive system to cure. Three Tygon tubes were used to construct each specimen, around 2.0 mm in diameter and 1 mm in height. It was necessary to photoactivate the resin/tube sets for 40 seconds after they were mounted on enamel and filled with IPS Empress Shade: A2 composite resin. The non-photoactivated specimens were coated with an aluminum sheet to prevent further polymerization. Any samples with air bubbles or obvious holes at contact were discarded using an optical microscope at 10× magnification. A Bard parker (BP) blade was used to remove the extra adhesive around the tubes.

Micro-shear test

ACME Engineers in India have developed an electronic and software-based universal testing machine. The micro-shear bond test was carried out using the UNITEST-10 model. The universal testing machine connected each PVC tube holding a bonded specimen to the testing apparatus. Each sample was exposed to a shear force of 1.0 mm/minute until failure occurred, with a blade positioned as close to the resin-enamel contact as possible. It was possible to compute the bond strength by dividing the failure force by the bound area.

Statistical analysis

To analyze the micro-shear bond inside all trial gatherings to all subgroups, the qualities obtained for every example were noted as the median value for factual examination, which was done utilizing SPSS version 20.0 (IBM Corp., Armonk, NY, USA) and the following accompanying tests: two-way analysis of variance (ANOVA) (example treatment versus rebuilding time point) and Tukey’s post-hoc test (p = 0.05).

## Results

The values of mSBS were statistically significant among all groups and subgroups with a p-value of 0.0001 (p < 0.05), as measured by the two-way ANOVA test. Mean mSBS (MPa) and the respective standard deviations, according to specimen treatments and restoration time points, are listed in Table 1.

**Table 1:** Mean and SD of micro-shear bond strength (MPa) in three groups (MI, MIF, FMI) and four subgroups (immediate, seven days, 14 days, 21 days). MI: McInnes; MIF: McInnes fluoride; FMI: fluoridated McInnes; SD: standard deviation

Groups with subgroups	N	Mean	SD	P-value
MI at immediate	10	11.92	2.40	0.00
MI at 7 days	10	16.19	3.45	0.01
MI at 14 days	10	20.82	0.88	0.00
MI at 21 days	10	21.34	1.32	0.01
MIF at immediate	10	10.40	2.30	0.03
MIF at 7 days	10	16.51	2.51	0.02
MIF at 14 days	10	19.55	1.18	0.00
MIF at 21 days	10	20.24	1.23	0.00
FMI at immediate	10	17.41	1.07	0.03
FMI at 7 days	10	19.34	1.13	0.02
FMI at 14 days	10	19.91	0.89	0.00
FMI at 21 days	10	20.81	0.87	0.00

Pair comparison of the three groups (MI, MIF, and FMI) and four subgroups (immediately, seven days, 14 days, and 21 days) with mSBS (MPa) by Tukey’s multiple post-hoc test was done where there was a statistically significant difference (p-value < 0.05) between all groups except (1) between the 14-day and 21-day restoration time point subgroups in all the three groups. (2) Between all four similar restoration time point subgroups in groups MI and MIF. (3) Between all four subgroups of group FMI, except FMI (immediate) and FMI (after 21 days) (Table 2).

**Table 2:** Various comparative measurements of the shear strength among groups. MI: McInnes; MIF: McInnes fluoride; FMI: fluoridated McInnes; SD: standard deviation; MPa: megaPascal

	Immediate restoration (values in MPa)	Restoration after 7 days ( values in MPa)	Restoration after 14 days (values in MPa)	Restoration after 21 days (values in MPa)
Group MI	11.92 ± 2.40	16.19 ± 3.45	20.82 ± 0.88	21.34 ± 1.32
Group MIF	10.40 ± 2.30	16.51 ± 2.51	19.55 ± 1.18	20.24 ± 0.81
Group FMI	17.41 ± 1.07	19.34 ± 1.13	19.91 ± 0.89	20.81 ± 0.81
Control group	22.74 ± 1.72			

Hence, the mSBS values obtained for all groups immediately and at seven days were lower, while at 14 and 21 days were similar to the control group. The mSBS results showed higher values for group FMI than groups MI and MIF immediately and at seven days. Therefore, using a fluoridated McInnes solution proved to be effective in immediately reversing the side effects of low mSBS when in-office bleaching is used.

## Discussion

Discoloration of the teeth has long been a major source of worry, especially in light of the recent rise in the demand for whiter smiles. Hydrochloric acid and hydrogen peroxide are often used to remove stains from the enamel or dentin of significant teeth [18]. The McInnes bleaching chemical was selected for this investigation because it is most often utilized in clinical settings to treat dental fluorosis. Because of the high oxidizing impact of hydrogen peroxide on enamel, intrinsic stains bleach teeth by altering the enamel matrix [7]. Its low molecular weight allows hydrogen peroxide to pass through enamel and decompose into water and oxygen free radicals, which interact with colored organic molecules and form stains. In the presence of free radicals, these vibrant macromolecules are reduced to simpler building blocks. Slightly lighter or colorless molecules with shorter wavelengths provide the whitening effect [7].

We began to wonder about the impact of bleaching procedures on tooth structure and the ensuing adhesive bond strength to bleached dental structures as the number of procedures increased. The molecular weight is really low. Bleached enamel retains hydrogen peroxide, changing the enamel matrix structure and resulting in insufficient adhesive polymerization due to residual oxygen free radicals [14]. Because it is a vigorous oxidizing agent, it easily denatures proteins while entering into interprismatic zones. It is possible that alterations in the ultrastructure of resin-enamel interactions as a consequence of these modifications are to blame [12]. Surface porosities and morphological alterations are caused by enamel bleaching [19], resulting in increased stress concentrations in load-bearing zones. This weak enamel, which has a low fracture toughness and a low microhardness, is prone to premature fractures [17].

Many studies have been done on how dental bleaching affects the adhesive strength of restoratives that adhere to the teeth. Resin bonding and polymerization are hindered by the presence of residual peroxide on the tooth surface, according to some researchers [17]. However, there are many who believe that bleaching is required to remove the protein and mineral composition of enamel's surface layers, which reduces its ability to adhere [12]. According to Lai et al., these are reversible modifications caused by a change in the redox potential of the enamel substance, rather than permanent structural changes [10]. There has been a lot of progress in tooth bleaching and efforts to increase remineralization in the previous few years.

Using alcohol to clean the enamel [14] and employing an adhesive containing organic solvents are two methods for minimizing the confirmed decreased tooth-resin bond strength [15]. Resin-enamel bond strengths may take up to a year to restore to levels found in unbleached enamel, according to several studies. According to various reports, the waiting time might range from 24 hours to four weeks [19]. Topical fluoride stimulates remineralization and prevents demineralization of dental hard tissues by forming a calcium fluoride coating. Consequently, the aim of this research was to determine if fluoride may assist in restoring the binding strength of McInnes-bleached enamel to resin-based composite.

Two types of fluoride delivery were used in this study: fluoride incorporated into the bleaching solution and a separate post-bleaching topical fluoride treatment. The fluoride McInnes solution was prepared by mixing powdered NaF into newly mixed McInnes solution at a concentration of 2 g% and applying it topically using Knutson’s approach. For the separate topical fluoride application, a commercially available APF gel was used. For this experiment, the shear mode of testing was selected over the tensile mode because it would better represent the stresses exerted on veneer restorations on the tooth [19]. Artificial saliva was employed to store the specimens in between the restorative cycles in this investigation because saliva is thought to aid in remineralization and eventual bond strength repair over a period of seven, 14, or 21 days. Consequently, values were taken for each Group at four time intervals: immediately after the bleaching treatment, seven days later, 14 days later, and 21 days later [19].

According to the results of this experiment, the mSBS values in group MI for the subgroup restored quickly after bleaching treatment were low [10]. Consequently, the null hypothesis has been partly rebutted. When bleaching treatments decrease the dental/adhesive contact, they may impact mSBS values and explain this micro-shear test result by changing the enamel’s morphology, such as causing porosity and losing its prismatic structure. These theories are backed by previous research that examined what kinds of problems may arise at the bleached dental substrate/resin-composite interface and discovered mostly problems with adherence [11].

When looking for a way to reduce the negative effects that bleaching has on teeth, we looked at two options, namely, applying fluoride directly to the teeth, or including it in the bleaching chemical. Few studies have examined fluoride’s ability to remineralize enamel after a bleaching procedure (either topically or in the bleaching gel’s composition) under the parameters outlined above. Both topical and fluoride bleaching gels were tested in this study [5,6,19].

Topical fluoride therapy was found to be unsuccessful in reversing the MIF group’s lower mSBS values following restoration. Partially rejecting the second null hypothesis, which required a 14-day delay until results were equivalent to those in the control group. Group MI, which did not get fluoride treatment, also showed a rise in mSBS levels after 14 days, suggesting that the restoration time point rather than fluoride treatment was responsible for the increase in mSBS levels. Group FMI preserved mSBS values higher than groups MI and MIF, as well as being comparable to those of the control group during all restoration time points, suggesting that part of the second null hypothesis has been accepted. Restored mSBS levels were likewise found in all groups after 14 days of preservation on artificial media. We may hypothesize, based on the data, that the McInnes bleaching agent affects the mSBS of resin-based composites to enamel by causing demineralization, which can be restored spontaneously if restoration is delayed 14 days. No matter how much fluoride was applied to the teeth after in-office bleaching, the poor bond strength values remained. Fluoridated McInnes solution restored bond strength better than topical fluoride or no fluoride during seven days of bleaching and after a seven-day gap without fluoride.

As a result, the study provides insight into how one may aspire to extend the longevity of post-bleaching composite veneer treatments when a simple chairside addition of NaF to the McInnes bleaching solution is not practical. These results should be interpreted with caution as just one fluoride concentration and fluoridated bleaching chemical were examined in this study. To confirm and improve these findings, more research with several fluoride in-office bleaching agents, different concentrations of NaF, and comparisons with other topical remineralization agents is needed.

A small number of samples were used in this study, which was conducted in vitro is one of the limitations of the study. Further investigation and analysis are required before it can be utilized in the broader population.

## Conclusions

Based on this study, it is speculated that the mSBS of enamel to resin-based composites is reduced by McInnes bleaching agent. This reduction in the mSBS value is found to be influenced by the restoration time point. It is found to increase spontaneously with time, reversing to a normal value within 14 days. Results showed that binding strength values increased when a fluoridated McInnes solution was used to bleach teeth immediately or seven days later compared to when fluoride was administered topically. Therefore, the mSBS values are influenced by the type of fluoride delivery.

## References

[1] Matis BA, Cochran MA, Eckert G, Carlson TJ (1998). The efficacy and safety of a 10% carbamide peroxide bleaching gel. Quintessence Int.

[2] Haywood VB (1997). Nightguard vital bleaching: current concepts and research. J Am Dent Assoc.

[3] Heymann HO (2005). Tooth whitening: facts and fallacies. Br Dent J.

[4] Marshall K, Berry TG, Woolum J (2010). Tooth whitening: current status. Compend Contin Educ Dent.

[5] Tay LY, Kose C, Herrera DR, Reis A, Loguercio AD (2012). Long-term efficacy of in-office and at-home bleaching: a 2-year double-blind randomized clinical trial. Am J Dent.

[6] Lewinstein I, Fuhrer N, Churaru N, Cardash H (2004). Effect of different peroxide bleaching regimens and subsequent fluoridation on the hardness of human enamel and dentin. J Prosthet Dent.

[7] Seghi RR, Denry I (1992). Effects of external bleaching on indentation and abrasion characteristics of human enamel in vitro. J Dent Res.

[8] Haywood VB (2000). Current status of nightguard vital bleaching. Compend Contin Educ Dent Suppl.

[9] Schmidt-Nielsen B (1946). The solubility of tooth substance in relation to the composition of saliva. Acta Odontol Scand.

[10] Lai SC, Tay FR, Cheung GS (2002). Reversal of compromised bonding in bleached enamel. J Dent Res.

[11] Wilson D, Xu C, Hong L, Wang Y (2009). Effects of different preparation procedures during tooth whitening on enamel bonding. J Mater Sci Mater Med.

[12] Barghi N, Godwin JM (1994). Reducing the adverse effect of bleaching on composite-enamel bond. J Esthet Dent.

[13] Perdigão J, Francci C, Swift EJ Jr, Ambrose WW, Lopes M (1998). Ultra-morphological study of the interaction of dental adhesives with carbamide peroxide-bleached enamel. Am J Dent.

[14] Bulut H, Kaya AD, Turkun M (2005). Tensile bond strength of brackets after antioxidant treatment on bleached teeth. Eur J Orthod.

[15] Cvitko E, Denehy GE, Swift EJ Jr, Pires JA (1991). Bond strength of composite resin to enamel bleached with carbamide peroxide. J Esthet Dent.

[16] Lopes GC, Bonissoni L, Baratieri LN, Vieira LC, Monteiro S Jr (2002). Effect of bleaching agents on the hardness and morphology of enamel. J Esthet Restor Dent.

[17] Araujo EM, Baratieri LN, Vieira LC, Ritter AV (2003). In situ effect of 10% carbamide peroxide on microhardness of human enamel: function of time. J Esthet Restor Dent.

[18] Skrtic D, Hailer AW, Takagi S, Antonucci JM, Eanes ED (1996). Quantitative assessment of the efficacy of amorphous calcium phosphate/methacrylate composites in remineralizing caries-like lesions artificially produced in bovine enamel. J Dent Res.

[19] Borges AB, Yui KC, D'Avila TC, Takahashi CL, Torres CR, Borges AL (2010). Influence of remineralizing gels on bleached enamel microhardness in different time intervals. Oper Dent.

